# Gellan Fluid Gel Embedded in Alginate Bead System: A New System for Encapsulation of *Limosilactobacillus reuteri* and Its Application in Sour Cherry Juice

**DOI:** 10.1002/fsn3.70199

**Published:** 2025-05-14

**Authors:** Zahra Najafpour, Mohammad‐Taghi Golmakani, Marzieh Moosavi‐Nasab, Seyed Mohammad Hashem Hosseini, Mehrdad Niakousari

**Affiliations:** ^1^ Department of Food Science and Technology, School of Agriculture Shiraz University Shiraz Iran

**Keywords:** alginate, gastrointestinal condition, gellan fluid gel, probiotic, sour cherry juice

## Abstract

In the present research, we aimed to create a novel controlled‐release device using two polymeric systems: beads of alginate hydrogel embedded with gellan fluid gel that contained *Limosilactobacillus reuteri* cells. Three different formulations, 1.5% alginate (Alg), 1.35%:0.15% alginate:gellan (Alg:Gel), and 1.2%:0.3% (Alg:Gel), were used in this research. We investigated the effect of this system on the physical properties of beads and probiotic viability, as well as application in sour cherry juice (for 8 weeks). The results showed that the presence of gellan fluid gel in the alginate microcapsules increased the hardness and diameter of the beads from 1.15 to 3.43 N and 1.71 to1.87 mm, respectively. Also, this system improved the viability of the microencapsulated bacteria under simulated gastrointestinal and thermal conditions (final counts of 7.17 and 7.00 logs CFU/mL, respectively). Regarding the probiotic viability during 28 days of storage (−18°C, 4°C, and 25°C), the counts of all treatments decreased during the storage, but the best results were observed at the freezing temperature (7.85 log CFU/mL). Raman confocal microscopy showed that the wall thickness of beads decreased by increasing gellan fluid gel from 10.3 to 8.6 μm. In the case of sour cherry juice, the viability of cells decreased in the range of 7.4–8.7 log CFU/mL, vitamin C in the range of 0.45–1.14 mg/L, and phenolic content in the range of 0.42–0.62 mg/L at the end of the storage period. As a result, the samples containing encapsulated *L. reuteri* , especially alginate/gellan fluid gel beads, showed better results. In summary, the beads made from 1.2% alginate and 0.3% gellan fluid gel proved to be an effective delivery and release system for *L. reuteri* cells and application in sour cherry juice.

## Graphical abstract

In the present study, we aimed to create a novel controlled‐release device using two polymeric systems: beads of alginate hydrogel embedded with gellan fluid gel that contained *Lactobacillus reuteri* cells. The results showed that alginate microcapsules with gellan fluid gel resulted in harder beads with larger particle size diameters and enhanced the viability of the microencapsulated bacteria under simulated gastrointestinal and thermal conditions. In summary, the beads made from 1.2% alginate and 0.3% gellan fluid gel proved to be an effective delivery and release system for *L. reuteri* cells and application in sour cherry juice.

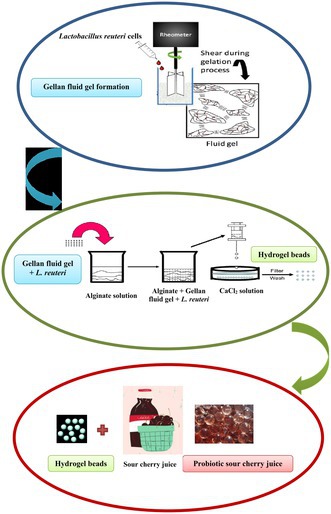

## 1 Introduction

Probiotics are beneficial microorganisms that provide some health benefits to the host if adequate amounts (10^7^ CFU/g) are consumed (Etchepare, Raddatz, Flores, et al. 2016). *Lactobacillus* spp. is a broad group of probiotics. *Lactobacillus reuteri* is one member of this group. *L. reuteri* can be found in some parts of the human body, such as the gastrointestinal tract, skin, and breast milk. This probiotic provides many beneficial effects for the host body (Mu et al. 2018). Adequate amounts of this probiotic must reach the colon (Zhao et al. 2012). Food is a suitable carrier for the consumption of probiotics, especially for children. Some studies have investigated the application of encapsulated probiotics in different food systems such as dairy products, fruit juices, jelly desserts, chocolate, etc. (Hruyia et al. 2018; Mokhtari et al. 2019; Rajaie Azarkhavarani et al. 2019; Sabbaghpour Langaroudi et al. 2023).

To adequate amounts of probiotics reach the colon, the probiotics should be protected from food processing and inappropriate conditions of the gastrointestinal tract (low pH of the stomach and high bile salt concentration of the small intestine) (Etchepare, Raddatz, Cichoski, et al. 2016). Encapsulation is a standard method to resolve this issue. Different materials can be used as encapsulating agents, such as alginate, gums, proteins, and even synthetic polymers like polyvinyl alcohol (PVA) (Kim et al. 2016). Among these materials, sodium alginate has been used frequently to encapsulate sensitive ingredients like probiotic microorganisms due to its good properties, such as forming a highly versatile, biocompatible, non‐toxic matrix, and easy gelling process. However, the alginate gel is porous and sensible to extreme pH values. There are different ways to overcome these drawbacks, and one way is combining alginate with other encapsulating agents or systems (Etchepare, Raddatz, Flores, et al. 2016).

Gellan gum is an anionic and extracellular hydrocolloid produced by *Pseudomonas elodea*. Gellan gum can be an encapsulating agent since it has good acid and heat tolerance. Also, gellan gum can form a fluid gel (Omoto et al. 2000). When a polymer solution is sheared while cooling and gelation are occurring, a fluid gel or sheared gel is formed. In a fluid gel system, despite bulk gel, particles are included within the solution so that fluid gel can be defined as a solution of gelled particles (Farrés et al. 2014; Bradbeer et al. 2015). Additionally, gellan gum is explicitly degraded in the colon by galactomannan enzymes. Briefly, the formation of fluid gel from low acyl gellan gum contains four steps: (a) dispersion, (b) hydration, (c) addition of salt or acid, and (d) cooling process (Imeson 2009). Some studies have investigated the formation and rheological characteristics of gellan fluid gel (Bradbeer et al. 2015; García et al. 2018). Studies about the application of gellan fluid gel as a carrier for the delivery of different ingredients are minimal. This application is more in the field of pharmaceutics and for delivery of components such as diclofenac (non‐oral use) (Mahdi et al. 2016) or spray delivery of cells (Ter Horst et al. 2019).

The present study was performed to design a new encapsulation and delivery system for the encapsulation of probiotics. This system combined gellan fluid gel and the alginate bead system. Then, the capability of this system was evaluated in a simulated gastrointestinal tract and sour cherry juice.

## 2 Materials and Methods

### 2.1 Materials

*Limosilactobacillus reuteri* (ATCC 23272) was supplied from the Persian Type Culture Collection. MRS broth and agar (ibresco, Karaj, Iran); calcium chloride, mono and di‐potassium phosphate, sodium hydroxide, and hydrochloric acid (from Merck Co., Darmstadt, Germany); sodium alginate (*M*_*w*_ of around 190 g mol^−1^, *M*/*G* = 1.5), gellan gum (*M*_*w*_ of around 500 kDa), bile salts, and pancreatin and pepsin enzymes (from Sigma‐Aldrich (St. Louis, MO)) were the main compounds that were used in this research.

### 2.2 Methods

#### 2.2.1 Culture Preparation

The frozen stock culture of *L. reuteri* was activated by inoculation in MRS broth for 48 h at 37°C. Then, the bacterial pellet was separated by centrifugation at 3200 *g* for 10 min at 4°C (Muthukumarasamy et al. 2006; Zhao et al. 2012). The resulting bacterial pellet was washed with sterile 0.9% NaCl solution (saline solution) and suspended in saline (concentration of 10^10^ CFU/mL) for the next steps.

#### 2.2.2 Sour Cherry Juice Preparation

Fresh sour cherry fruit ( *Prunus cerasus* ) was purchased from the local market in Shiraz, Iran. Briefly, the fruits were washed and compressed for sour cherry juice production. The obtained sour cherry juice was centrifuged for the removal of additional compounds. Then, the prepared juice was evaluated as a vehicle for supplying probiotics (Arjeh et al. 2015).

#### 2.2.3 Optimization of Entrapment Substances for Probiotic Encapsulation

##### 2.2.3.1 Formation of Gellan Fluid Gel

Gellan fluid gel was prepared based on the method used by Garcı'a et al. (2015) with some modifications. In the first step, gellan gum powder (0.15 and 0.3 g) was added slowly to the hot deionized water (to reach a final weight of 50 g) located on a hot plate stirrer under continuous agitation (700 rpm). The hydration was achieved by keeping the solution for 25 min at 80°C. In the second step, the hot gellan gum solution was cooled under gentle agitation at room temperature until 38°C–40°C. Then, 1 mL of the bacterial suspension was added to the gellan gum solution. In the third and fourth steps, gellan solution containing bacterial suspension was agitated and sheared by an Ultra‐Turrax homogenizer system (IKA, T18, Germany) under an appropriate shear rate (3000–4000 rpm). The required amount of CaCl_2_ solution was added slowly by a syringe and the solution was sheared and cooled for about 5 min. After this time, a homogenous gellan fluid gel containing *L. reuteri* cells was made (García et al. 2015).

##### 2.2.3.2 Bead Formation

A simple extrusion method was used for the bead formation. The gellan fluid gels that contained *L. reuteri* cells from the previous step were mixed with different concentrations of alginate solution (1.35 and 1.2 g alginate in deionized water with a total weight of 50 g) at 40°C–45°C. After combining these systems, we had a solution with a final concentration of 1.5% (w/w) dry matter. After obtaining a homogeneous system, this mixture was dripped by a plastic syringe (22 Gauge Needle) into 1.5% (w/v) CaCl_2_ solution. The formed beads remained for 30 min in this hardening solution and were then harvested and washed with deionized water (Lotfipour et al. 2012). The solution of 1.5% (w/w) alginate that contained *L. reuteri* cells was used as a control.

#### 2.2.4 Physical Properties of Beads

##### 2.2.4.1 Size and Morphology

The average diameter of beads was measured by micrometer (Nualkaekul et al. 2012). Scanning electron microscopy (SEM) (TESCAN vega3, Czech Republic) was used to investigate the morphological properties of the beads, based on the method employed by Khajehie et al. (2023).

##### 2.2.4.2 Hardness

The hardness of the beads was evaluated by the method employed by Sandoval‐Castilla et al. (2010). The beads were compressed using two cycles (30% compression and a constant crosshead velocity of 0.5 mm/s) (Brookfield Engineering Labs Inc., TexturePro CT V1.3, America). The hardness was calculated based on the maximum required force (N) for bead compression (Sandoval‐Castilla et al. 2010; Khajehie et al. 2023).

##### 2.2.4.3 Raman Confocal Microscopy

We used Raman confocal microscopy (LabRAM HR, Horiba, Japan) and point‐by‐point mapping method to get some information about the wall thickness of alginate and alginate/gellan fluid beads, according to the method described by Khajehie et al. (2023) (Rahiminezhad et al. 2020).

#### 2.2.5 Microbial Properties of Beads

##### 2.2.5.1 Encapsulation Efficiency

Encapsulation efficiency (EE) can be explained as the concentration of the incorporated material in the formed beads over the initial concentration used for making the beads. EE of *L. reuteri* was determined using the method described by Ganje et al. (2024).

(1) $$\text{Encapsulation efficiency}\;\left(\%\right)=\left({\mathrm{Log}}_{10}\;N/{\mathrm{Log}}_{10}\;N_{0}\right)\times 100$$

In this equation, *N* is the viable CFUs (Colony Forming Units) after encapsulation, and *N*_0_ represents the viable CFUs before encapsulation.

##### 2.2.5.2 Survival of Encapsulated *L. reuteri* in Different Simulated Gastrointestinal Conditions

In this study, the survivability of free and encapsulated *L. reuteri* was investigated according to the method employed by Haghshenas et al. (2015) in three different conditions. One gram of fresh beads or 1 mL of free cell suspension was inoculated into 9 mL of simulated gastric fluid (9 g/L of NaCl, 3 g/L of pepsin, and pH 1.8 at 37°C for 120 min), followed by a placement in simulated intestinal fluid (9 g/L of NaCl, 3 g/L of bile salts, 10 g/L of pancreatin, and pH 6.5 at 37°C for 120 min). Finally, the treated beads were separated from the intestinal solution and transferred to the simulated colonic condition (0.1 M monobasic potassium and pH 7.4 at 37°C for 240 min). Counting was performed after 30, 60, and 120 min of gastric condition, 30, 60, and 120 min of intestinal condition, and 30, 60, 120, 180, and 240 min of colonic condition (Haghshenas et al. 2015; Etchepare, Raddatz, Cichoski, et al. 2016).

##### 2.2.5.3 Thermal Treatment

The heat resistance of free and encapsulated *L. reuteri* was investigated according to the method described by Raddatz et al. (2022) with some modifications in two thermal conditions: 63°C for 30 min and 75°C for 3 min.

##### 2.2.5.4 Survival of Encapsulated L. reuteri in the Storage Conditions

Free and encapsulated *L. reuteri* were stored in saline solution at −18°C, 4°C, and 25°C for 28 days, and the storage ability of cells was evaluated at 0, 7, 14, 21, and 28 days of storage (Dafe et al. 2017).

#### 2.2.6 Incorporation of Encapsulated *L. reuteri* in Sour Cherry Juice

Incorporating free and encapsulated *L. reuteri* in sour cherry juice was performed based on the method employed by da Marques Silva et al. (2021). Briefly, 10 mL of pasteurized sour cherry juice was transferred to sterile capped tubes, along with the addition of 1 g of encapsulated probiotic or 1 mL of free cells, followed by storage at 4°C. Thus, there were three treatments in this part of the study: free cells, microcapsules with 1.5% alginate (control), and beads from the best formulation of alginate and gellan fluid gel (da Marques Silva et al. 2021).

##### 2.2.6.1 Probiotic Viability During Storage for 8 Weeks at 4°C

Free and encapsulated probiotics (alginate and alginate/gellan fluid gel beads) were stored in sour cherry juice for 8 weeks at 4°C. The viability of cells was analyzed weekly (da Marques Silva et al. 2021).

##### 2.2.6.2 Physicochemical Properties of Sour Cherry Juice

The pH (with a table‐type pH meter, OHAUS, Starter 2000, USA), acidity (by titration), total soluble solids (TSS) (with a refractometer, CARL ZEISS, Germany), and color of probiotic sour cherry juices were analyzed weekly for 8 weeks, according to Yildiz et al. (2022).

##### 2.2.6.3 Total Phenolic Content and Radical Scavenging Activity

The total phenolic content (TPC) and radical scavenging activity (RSA) of samples were determined based on the method used by Yildiz et al. (2022) after 0, 4, and 8 weeks of storage. TPC is measured as gallic acid equivalent (GAE), and the RSA of sour cherry juice samples was calculated based on the DPPH radical scavenging ability according to the following equation:

(2) $$\text{Radical scavenging activity}\;\left(\%\right)=\left(\left(A_{\text{control}}–A_{\text{sample}}\right)/A_{\text{control}}\right)\times 100$$

##### 2.2.6.4 Vitamin C Content

The vitamin C content of different sour cherry juice treatments was measured by HPLC (Smartline, Knauer Company, Berlin, Germany), according to the method described by Furusawa (2001), after 0, 4, and 8 weeks of storage.

##### 2.2.6.5 SEM Analysis of Beads in Sour Cherry Juice

SEM analysis was done to investigate the effect of sour cherry juice on the morphological characteristics of beads after 8 weeks of storage, according to the method described by Khajehie et al. (2023).

#### 2.2.7 Statistical Analysis

The experimental data in this study were analyzed using SPSS 16.0 (SPSS Inc., Chicago, IL) software (One‐way analysis of variance (ANOVA) and Duncan's multiple range tests). All sample analyses were performed in triplicate, and the values were presented as the means ± standard deviation (SD) (Dafe et al. 2017).

## 3 Results and Discussion

### 3.1 Physical Properties of Beads

#### 3.1.1 Diameter and Hardness of Beads

According to the data reported in Table 1, the use of gellan fluid gel in the microencapsulation significantly (*p <* 0.05) increased the diameter of the beads. Alginate beads had a mean diameter of 1.71 mm, while adding gellan fluid gel to the beads increased the diameter to 1.87 mm. This variation in the size of beads can be related to different polymer concentrations and compositions (Voo et al. 2011). Adding gellan fluid gel into the alginate solution produced a mixture with higher viscosity. Thus, alginate/gellan fluid gel formulations made the beads with larger sizes and uniform shapes. The results of our study were consistent with other researchers who expressed that decreasing the viscosity of supporting gels in the extrusion method led to smaller beads (Lotfipour et al. 2012; Haghshenas et al. 2015). The Larger beads have less specific surface area; thus, they can provide more protection against harsh conditions.

**TABLE 1:** Diameter, hardness, and encapsulation efficiency of beads.

Sample	Diameter (mm)	Hardness (*N*)	Encapsulation efficiency (%)
Alginate (1.5%)	1.71 ± 0.08^a^	1.15 ± 0.38^a^	78.90 ± 1.55^a^
Alginate:Gellan** (1.35%:0.15%)	1.82 ± 0.06^b^	1.70 ± 0.18^a^	86.64 ± 2.31^b^
Alginate:Gellan (1.2%:0.3%)	1.87 ± 0.10^b^	3.43 ± 0.01^b^	78.55 ± 3.36^a^

*Note:* Different superscript letters in each column indicate that the means differ significantly (*p <* 0.05). Values are the mean ± SD (*n* = 3).

** Gellan in the form of gellan fluid gel.

The composition and concentration of biopolymers affect the hardness of beads. The hardness of alginate beads was 1.15 N, while the gellan fluid gel addition significantly (*p <* 0.05) increased the hardness of the beads to 3.43 N (Table 1). Results showed that differences in the formulation led to different mechanical responses to the deformation. Moreover, it may be inferred that varying the ratios of alginate and gellan fluid gel in the formulation leads to beads with different textural and physical properties (Sandoval‐Castilla et al. 2010; Bepeyeva et al. 2017). The hardness is an important physical property that affects the bead stability during storage and within a food product. The presence of low acyl gellan gum leads to the formation of a hard gel. This gel is more resistant to digestive conditions due to its high resistance to heat, salt, and acid (Pirsa and Hafezi 2023).

#### 3.1.2 Confocal Raman Spectroscopy of Beads

**FIGURE 1:**
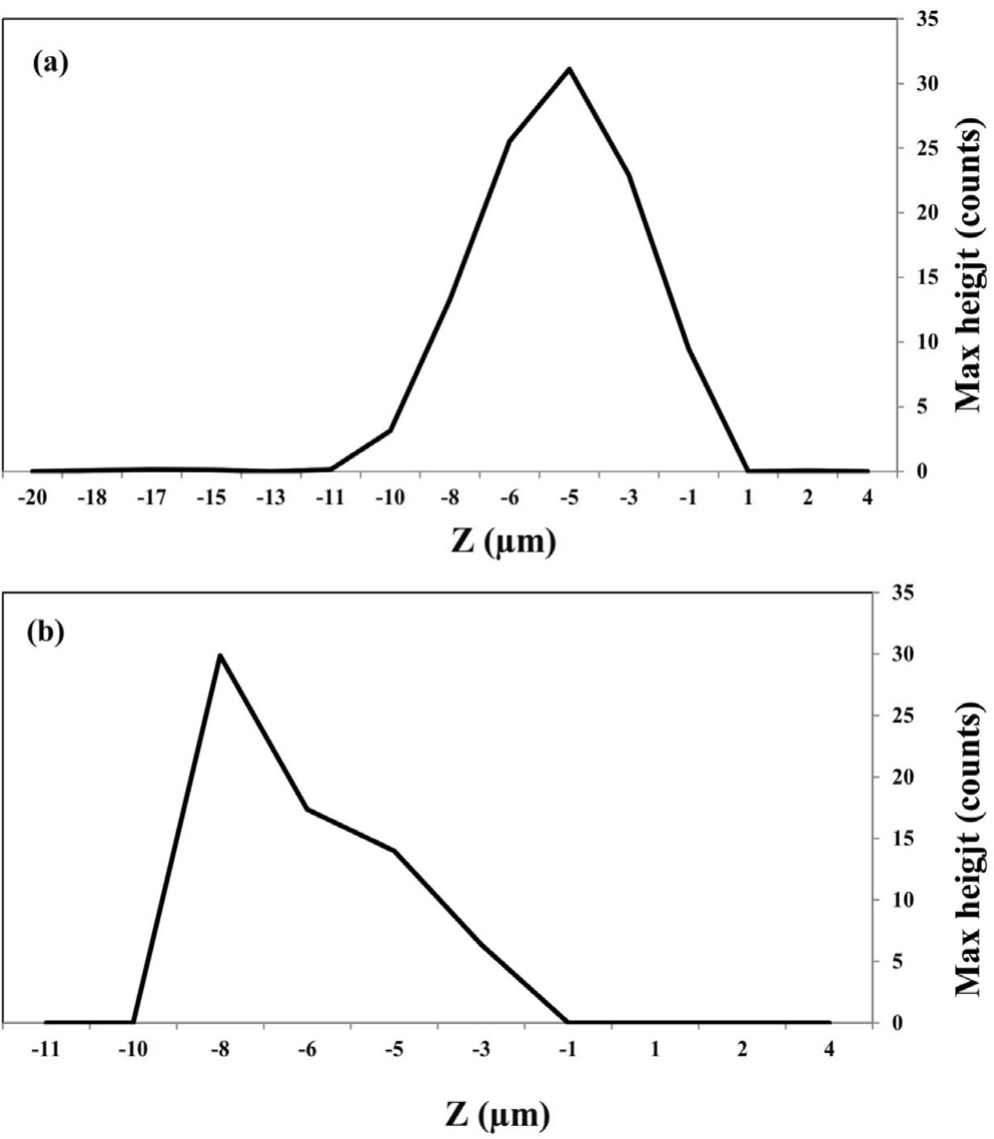
Red map Z‐profile in 860–900 cm^−1^, (a) alginate (1.5%, w/w) and (b) alginate:gellan fluid gel (1.2%:0.3%, w/w) beads.

The wall thickness of different beads is shown in Figure 1. Results revealed that the thickness of the wall was 10.3 and 8.6 μm for alginate (1.5%) and alginate:gellan fluid gel (1.2%:0.3%), respectively (based on calcium alginate reference Raman shift = 860–900 cm^−1^). The lower wall thickness of alginate:gellan fluid gel beads can be related to the lower alginate concentration as a bead‐forming agent. The evidence in this study was agreed with those explained by Khajehie et al. (2023). They investigated the wall thickness of different hydrogel beads (alginate, gellan, and gelatin) by Raman confocal microscopy and expressed that the wall thickness of samples varied between 5 and 6 μm.

### 3.2 Microbial Properties of Beads

#### 3.2.1 Encapsulation Efficiency

The EE of *L. reuteri* in the beads was in the range of 78.55% and 86.64%. According to Table 1, the 1.5% alginate beads showed significantly (*p <* 0.05) lower EE (78.90%) compared to the formulation that contained 1.35%:0.15% of Alg:Gel (86.64%). The alginate particles are porous, so combining alginate with other biopolymers can improve particle structure and enhance the EE of the probiotics. Also, the formulation that contained a high concentration of gellan fluid gel (1.2%:0.3% of Alg:Gel) showed low EE (78.55%) due to the high viscosity and less cell entrapment. Many researchers have expressed that using hydrocolloids can improve the porous structure of alginate beads (Azam et al. 2022). Rodrigues et al. (2020) used different mucilages and gums in combination with alginate to microencapsulate *Limosilactobacillus reuteri*. They reported that using these compounds combined with alginate enhanced the EE by improving the porous structure of the alginate bead.

#### 3.2.2 Survival of Encapsulated *L. reuteri* Under Simulated Gastrointestinal Condition

**FIGURE 2:**
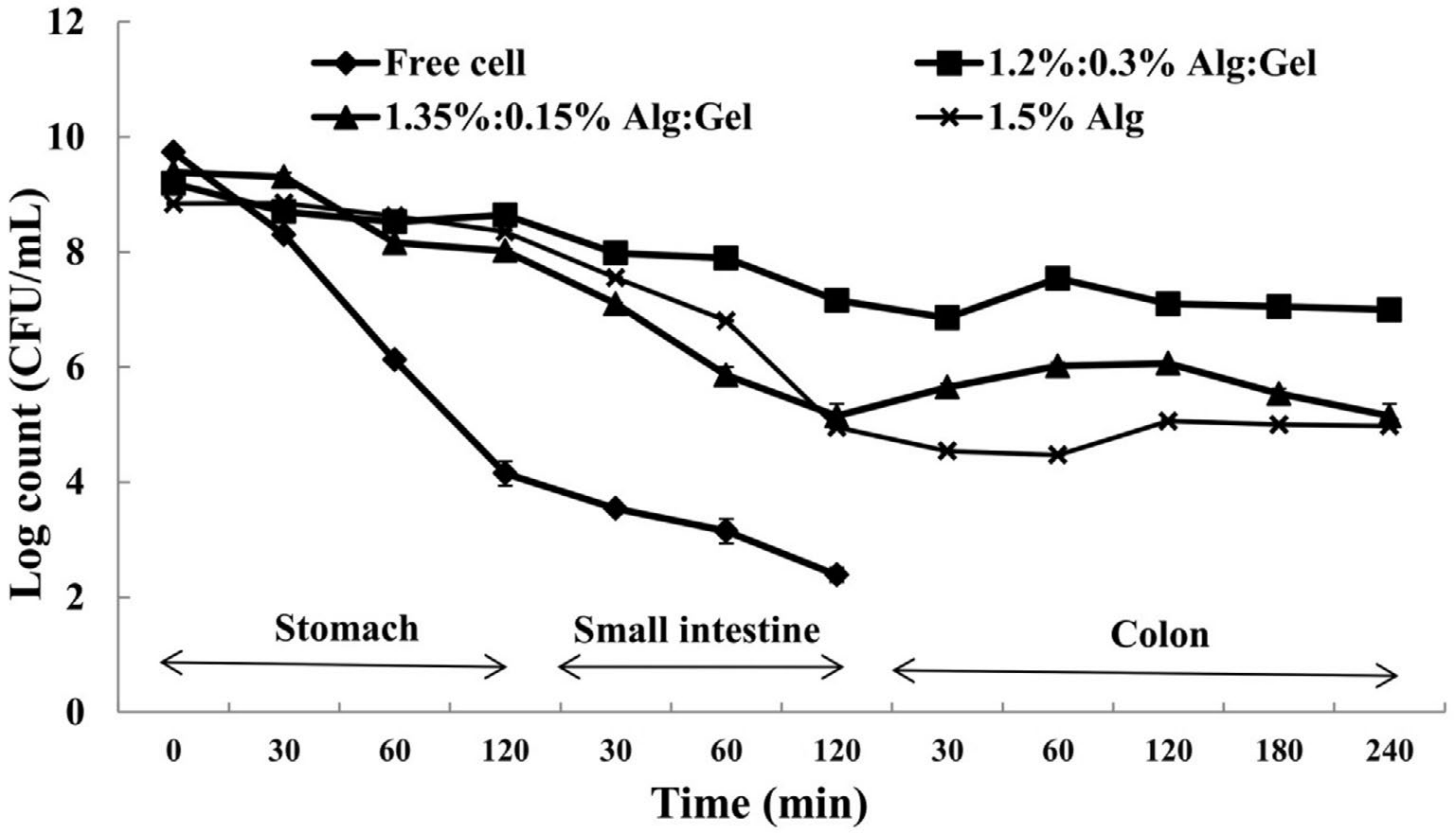
Survival of free and encapsulated *Limosilactobacillus reuteri* (log CFU/mL) in different simulated gastrointestinal conditions (Alg, alginate; Gel, gellan fluid gel). Mean ± SD (*n* = 3); Statistical test: ANOVA and multiple comparison of means using Duncan's multiple range test (*p* < 0.05).

The survivability of free and encapsulated *L. reuteri* in simulated gastric (120 min), intestinal (120 min), and colonic fluid (240 min, only for treated beads) is shown in Figure 2. The total counts of free *L. reuteri* declined approximately 5 log CFU/mL in simulated gastric fluid and 2 log CFU/mL in simulated intestinal fluid (totalizing 7 log CFU/mL). These results showed that *L. reuteri* cells were sensitive to simulated gastrointestinal conditions, confirming the necessity of encapsulating this microorganism. All treated beads were resistant to acidic pH, and no significant differences were observed. The beads shrank in gastric fluid. The low pH of gastric fluid resulted in protonation and decreased the electrostatic repulsive forces. The calcium ions were dissociated from the beads structure, and acidic beads were formed (Rayment et al. 2009). The addition of gellan fluid gel increased bacteria resistance not only to the low pH of simulated gastric fluid but also to high bile salts concentration in the simulated intestinal conditions. The formulation of 1.2%:0.3% (Alg:Gel) significantly increased the resistance of beads in the simulated intestinal fluid. In this case, approximately 1.5 log reduction was observed in comparison with other formulations (around 3 log reduction for 1.5% Alg and 1.35%:015% of Alg:Gel). In the intestinal fluid, the pH rose to 6.5, and beads swelled due to the increase in electrostatic repulsive forces. Thus, the bead porosity and the contact of encapsulated cells with harsh surrounding media (bile salt) increased (Rayment et al. 2009). The greater protective ability of alginate/gellan fluid gel can be attributed to the dense beads formed by the strong structure of alginate and gellan fluid gel and the increased resistance of gellan gum to salt conditions. Also, Rodrigues et al. (2020) expressed that the encapsulation process protected the *L. reuteri* cells against the gastrointestinal environment. This effect was related to the existence of different gums in the structure of the beads. Khajehie et al. (2023) investigated the properties of varying hydrogel beads made by direct and reverse spherification. They expressed that gellan gum in alginate beads increased cell protection under gastrointestinal conditions.

After the previous steps, the treated beads were contacted to the colonic solution for 4 h, and the cell release was investigated after 30, 60, 120, 180, and 240 min of incubation. As shown in Figure 2, the release time of cells depends on the coating material. At first, alginate and gellan fluid gel layers declined the cell release, and a little cell release was observed during the first 30 min. After 30 min, the beads started to release. The most cell release was recorded after 2 h of incubation for all beads (5.08, 6.30, and 7.53 log CFU/mL for alginate (1.5%), alginate:gellan (1.35%:0.15%), and alginate:gellan (1.2%:0.3%), respectively). The higher pH of colonic fluid (7.4) increased bead swelling, and monobasic potassium phosphate accepted the Ca^2+^ from beads. Thus, the beads were disintegrated, and complete cell releases were achieved. Haghshenas et al. (2015) used a mixture of inulin, fenugreek, and alginate‐psyllium to microencapsulate *Enterococcus durans* . They investigated the cell release from beads in the colonic condition and reported that a complete release of cells was observed after 2 h of incubation.

#### 3.2.3 Effect of Heat Treatment on the Viability of *L. reuteri*

**FIGURE 3:**
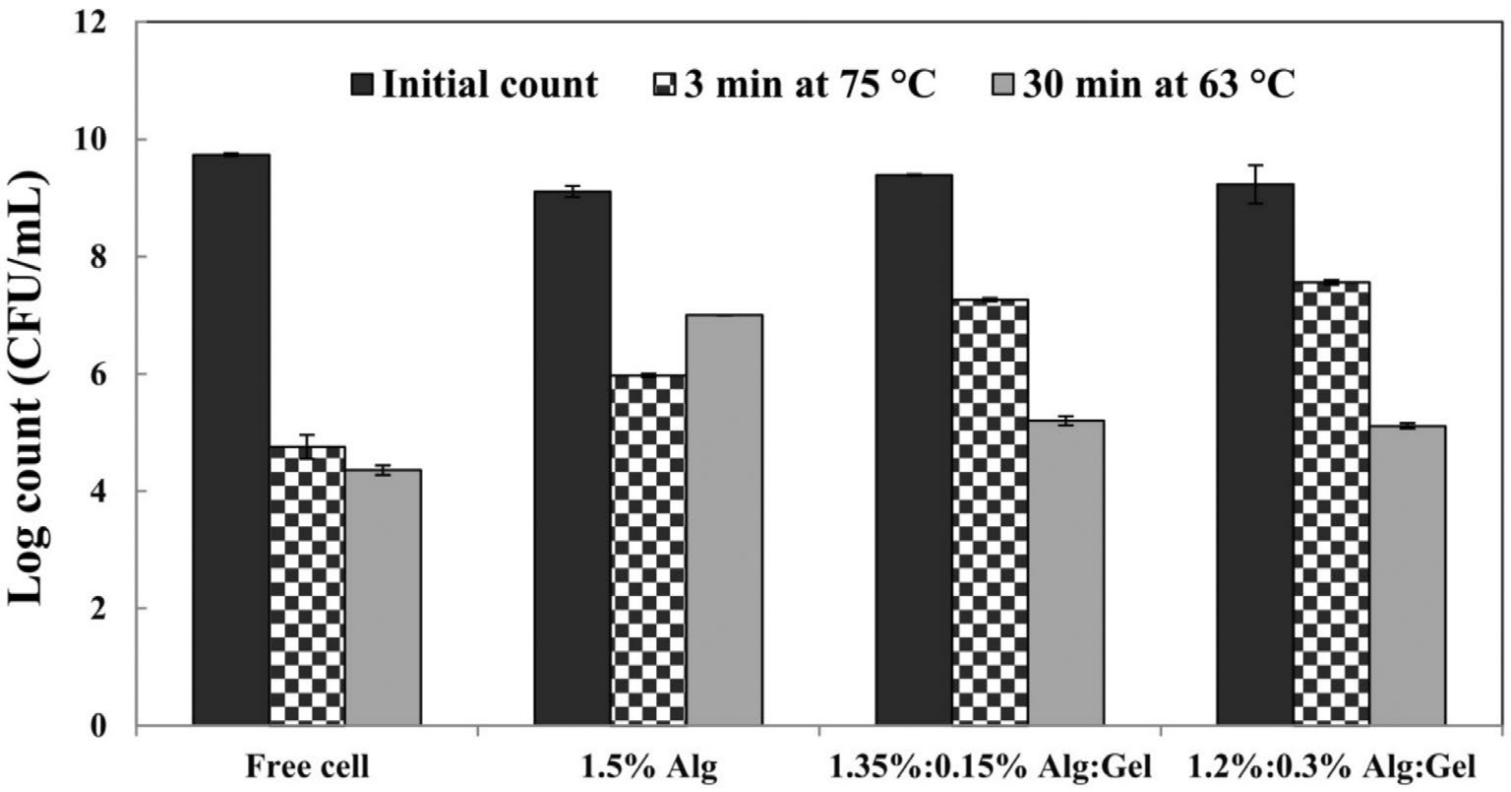
Effect of heat treatment on viable counts of free and encapsulated *Limosilactobacillus reuteri* (log CFU/mL) (Alg, alginate; Gel, gellan fluid gel). Mean ± SD (*n* = 3); Statistical test: ANOVA and multiple comparison of means using Duncan's multiple range test (*p* < 0.05).

The viability of probiotics under high‐temperature conditions depends on time and temperature (Avila‐Reyes et al. 2014). In this study, the thermal resistance of free and encapsulated *L. reuteri* was investigated at 63°C for 30 min and 75°C for 3 min (Figure 3). Data revealed that free cells were sensitive to high‐temperature conditions. At the same time, encapsulation significantly (*p* < 0.05) improved the heat tolerance of *L. reuteri* . The thermal treatment significantly decreased the counts of free cells from 9.75 to 4.41 and 4.90 log CFU/mL at 63°C for 30 min and 73°C for 3 min, respectively. Our results showed that alginate and alginate/gellan fluid gel, as the coating material, effectively protected *L. reuteri* cells from thermal treatment since the porous structure of beads works as a thermal protector. The beads that contained only 1.5% alginate were more resistant to low temperature and long time (63°C for 30 min) and the cell counts decreased from 9.17 to 7.00 log CFU/mL. But alginate/gellan fluid gel beads were more resistant to high temperature and short time (75°C for 3 min) since the heat resistance of gellan gum is more than alginate. The cell counts decreased from 9.46 to 7.59 log CFU/mL and 9.38 to 7.44 log CFU/mL for 1.35%:0.15% of Alg:Gel and 1.2%:0.3% of Alg:Gel formulations, respectively. Similarly, Youssef et al. (2020) encapsulated *L. salivarius* cells with a multilayer system and expressed that this system improved the heat resistance of cells (Youssef et al. 2020).

#### 3.2.4 Viability of Encapsulated *L. reuteri* During the Storage Period

**FIGURE 4:**
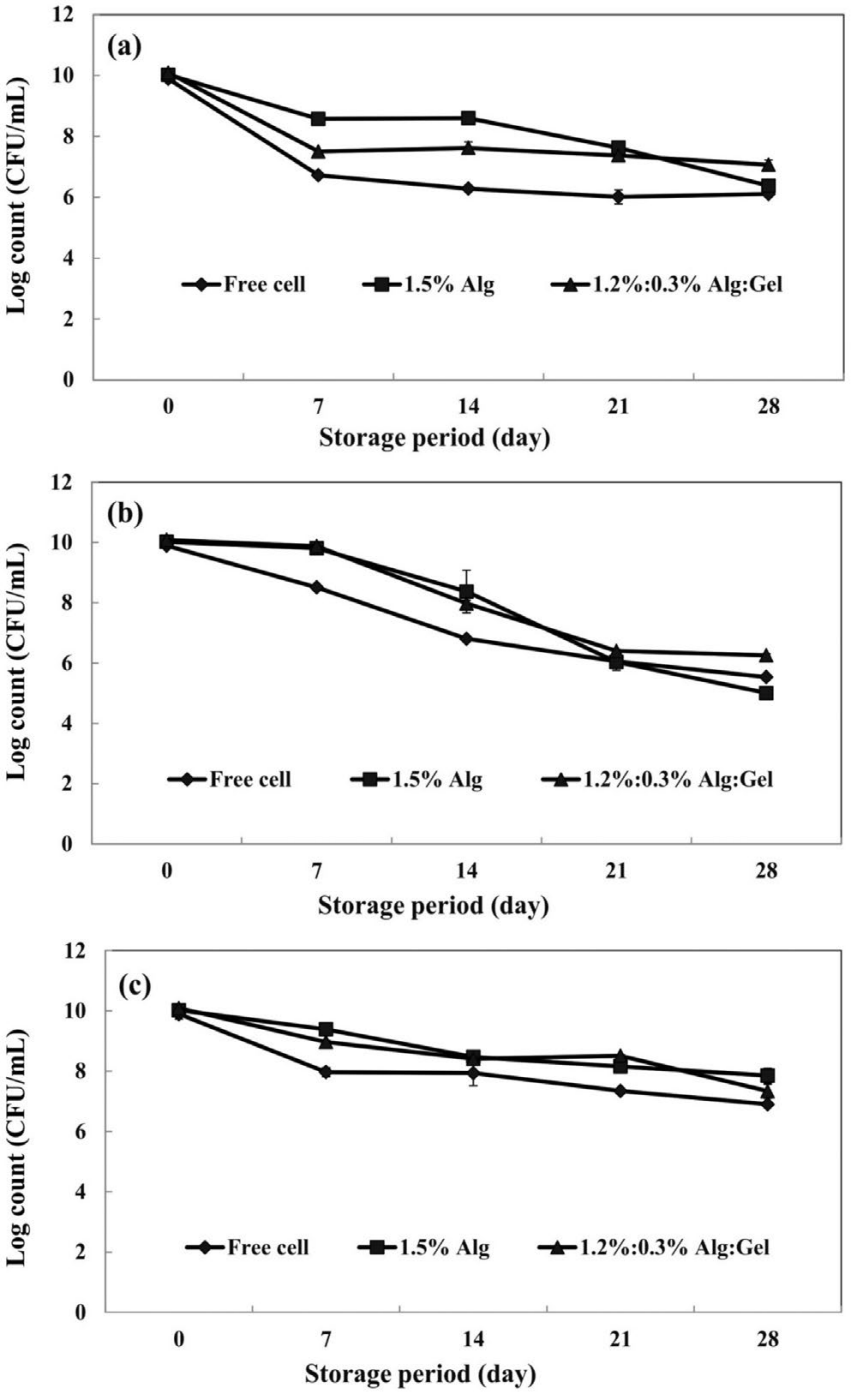
Survival of free and encapsulated *Limosilactobacillus reuteri* (log CFU/mL) during storage at different temperatures: (a) 4°C, (b) 25°C, and (c) −18°C (Alg, alginate; Gel, gellan fluid gel). Mean ± SD (*n* = 3); Statistical test: ANOVA and multiple comparisons of means using Duncan's multiple range test (*p* < 0.05).

The viability of probiotics decreases when they are stored (Cruz et al. 2010). So, we evaluated the influence of encapsulation on the survivability of *L. reuteri* under different storage temperatures. Our results revealed that the counts of all treatments significantly declined during the storage period, but the final counts of treated beads were almost suitable for conferring probiotic effects (6 log CFU/mL). Encapsulated *L. reuteri* in alginate and alginate/gellan fluid gel beads had higher viable counts during the storage period (Figure 4). The most viable cells were observed in the case of alginate/gellan fluid gel beads. Gellan fluid gel in beads provided a dense environment that effectively protected the bacteria under unsuitable conditions such as low temperature and long‐time storage.

Figure 4a shows the viability of free and encapsulated *L. reuteri* during 28 days of storage at 4°C. A rapid and significant decrease in viable cells was observed after 7 days for all treatments. In the case of free cells and alginate beads, there was about a 4 log reduction in viable cells. For alginate/gellan fluid gel, the reduction was lower (about 3 log) after 28 days of storage. Concerning room temperature (25°C), a significant decline in viability was recorded for all treatments after 28 days (Figure 4b). The best results for the survivability of *L. reuteri* were observed in frozen storage (−18°C). The viability of free cells decreased from10.09 log to 6.89 log after 28 days of frozen storage, while the beads of alginate and alginate/gellan fluid gel significantly (*p* < 0.05) remained more viable (7.85 and 7.33 log CFU/mL, respectively) during this period (Figure 4c). The decline in bacterial counts during frozen storage is related to injuries such as the formation of ice crystals and structural damage to the cell membrane (Homayouni et al. 2008). Our results were similar to those expressed by Etchepare et al. (2020) and Rodrigues et al. (2020). They declared that encapsulation of probiotics and freezing temperature efficiently improved the probiotic's viability compared to free cells.

According to the results of previous steps, the beads made of 1.2%:0.3% (Alg:Gel) were selected as the best formulation. So, 1.2%:0.3% (Alg:Gel) and 1.5% (Alg) beads and free cells were incorporated into the sour cherry juice for further steps.

### 3.3 Sour Cherry Juice

#### 3.3.1 Physicochemical Properties of Sour Cherry Juice During Storage Period

**FIGURE 5:**
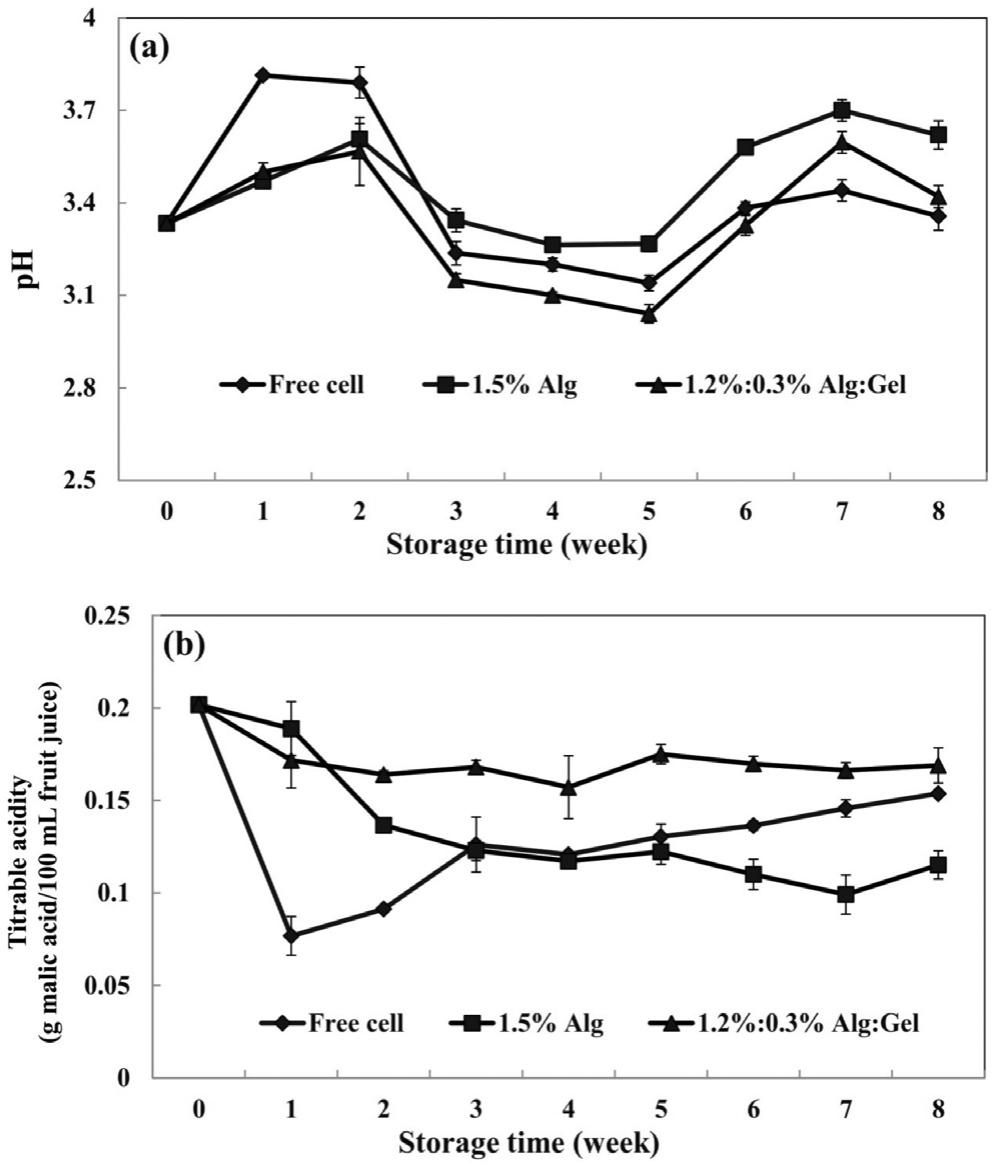
The variation of (a) pH and (b) titratable acidity of different sour cherry juice samples (Alg, alginate; Gel, gellan fluid gel). Mean ± SD (*n* = 3); Statistical test: ANOVA and multiple comparison of means using Duncan's multiple range test (*p* < 0.05).

**FIGURE 6:**
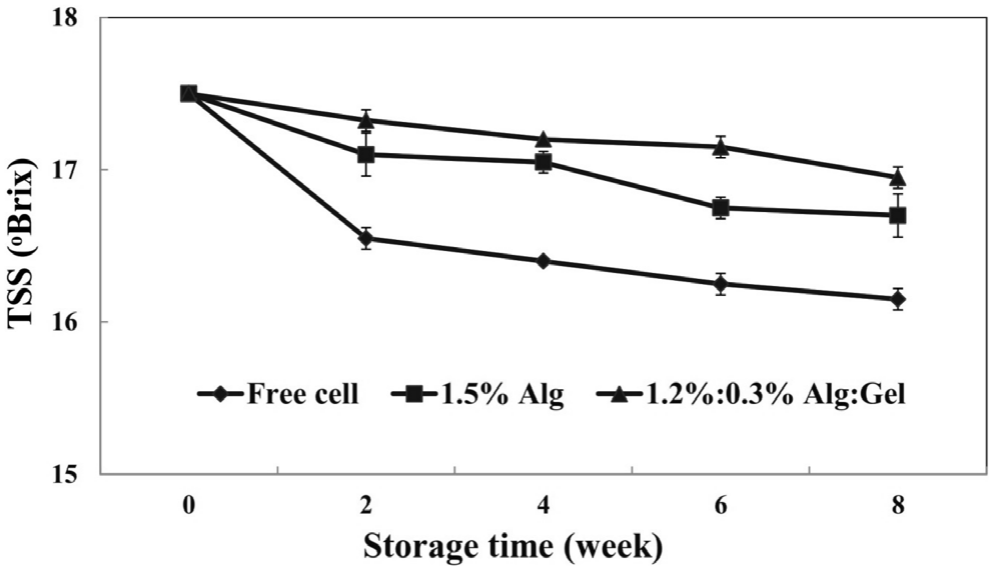
The variation of total soluble solid (TSS) in different sour cherry juice samples (Alg, alginate; Gel, gellan fluid gel). Mean ± SD (*n* = 3); Statistical test: ANOVA and multiple comparisons of means using Duncan's multiple range test (*p* < 0.05).

The fresh sour cherry juice showed pH (3.33), titratable acidity (0.2 g maleic acid/100 g fruit juice), and TSS (17.5°Brix) that were like those expressed by Yildiz et al. (2022). The results of this section are shown in Table 2, Figures 5 and 6. Probiotic bacteria reduce the pH of media by fermenting sugars and producing organic acids (Sohail et al. 2012). The pH of sour cherry juices changed in the range of 3.3–4.0. The pH of samples declined during the storage period, generally. In the first 5 weeks, the pH of samples decreased due to the fermentation of sour cherry juice sugars by *L. reuteri* cells. After this time, the sugar content of sour cherry juice was finished and the pH of samples increased slowly. The most significant pH variations were observed in the case of free cells from 3.33 to 3.62 (Figure 5a). Also, Rodrigues et al. (2020) expressed that the pH values of fruit juices changed during storage due to the presence of sugars, which can be fermented by free and microencapsulated *L. acidophilus* . Variation of titratable acidity confirms this issue. The titratable acidity of samples during 8 weeks of storage is shown in Figure 5b. The sour cherry juices that contained free cells had the most variations in titratable acidity, while less variation was observed in the case of alginate/gellan fluid gel beads. A reduction in TSS was observed in all samples, especially for free cells (Figure 6), which can be related to the fermentation of sugars in sour cherry juice by *L. reuteri* . Hruyia et al. (2018) reported that adding different species of *Lactobacillus* in sweet orange juice led to a significant reduction in the TSS of samples. Among the various parameters of our study, the most significant changes were observed in the case of free cells, indicating that microencapsulation (especially alginate/gellan fluid gel beads) efficiently reduced the contact of probiotics with the surrounding media.

**TABLE 2:** The color attributes of different sour cherry juice samples.

Color attribute	Storage time (week)				
	0	2	4	6	8
L*					
Free cell		11.6 ± 0.6^b^	13.0 ± 1.0^b^	15.7 ± 0.6^c^	22.7 ± 0.6^d^
Alg (1.5%)	10.0 ± 1.0^a^	13.6 ± 0.6^b^	18.7 ± 0.6^c^	20.3 ± 0.6^d^	19.0 ± 1.0^c^
Alg:Gel (1.2%:0.3%)		12.6 ± 0.6^b^	14.7 ± 0.6^c^	15.0 ± 1.0^c^	17.7 ± 0.6^d^
a*					
Free cell		31. 7 ± 0.6^b^	32.0 ± 1.0^b^	33.0 ± 1.0^b^	35.7 ± 1.1^c^
Alg (1.5%)	27.7 ± 0.6^a^	32.7 ± 0.6^b^	37.3 ± 1.1^d^	37.7 ± 0.6^d^	35.0 ± 1.7^c^
Alg:Gel (1.2%:0.3%)		34.0 ± 1.0^c^	33.3 ± 0.6^c^	36.3 ± 0.6^d^	29.7 ± 1.1^b^
b*					
Free cell		16.3 ± 1.1^b^	18.3 ± 1.5^c^	23.0 ± 1.0^d^	28.0 ± 1.0^e^
Alg (1.5%)	11.0 ± 0.0^a^	20.3 ± 1.1^b^	28.7 ± 0.6^cd^	30.0 ± 1.0^d^	27.3 ± 1.5^c^
Alg:Gel (1.2%:0.3%)		20.7 ± 0.6^c^	22.3 ± 0.6^d^	26.3 ± 0.6^e^	18.0 ± 1.7^b^

*Note:* Different superscript letters in each row indicate that the means differ significantly (*p <* 0.05). Values are the mean ± SD (*n* = 3).

Abbreviations: Alg, alginate; Gel, gellan fluid gel.

L*, a*, and b* values of samples are shown in Table 2. The color parameters of sour cherry juice on the first day of storage were like those expressed by Yildiz et al. (2022), who investigated the influence of some treatments on the physicochemical characteristics of homemade sour cherry juice. The color changes of the free cells containing the sample were more significant than other samples. Color analysis of samples showed that the L*, a*, and b* parameters generally increased during the storage period. This finding revealed that the color of samples changed to more brightness, redness, and yellowness over time. This phenomenon can be related to the decline in anthocyanin content of sour cherry juice over time, as well as the degradation of it by *L. reuteri* cells. Pilando et al. (1985) expressed a significant negative relation between the color parameters and total anthocyanin of strawberry wine. They expressed that increased L* and b* resulted from anthocyanin reduction during storage. Also, Gumus and Demirci (2022) investigated the survivability of some probiotics in grape juice and the physicochemical properties of the samples during cold storage. They expressed that added probiotic cultures into grape juice are active, so they degrade the pigments such as carotenoids in grape juice.

#### 3.3.2 Total Phenolic Content and Radical Scavenging Activity

TPC and RSA of samples are shown in Table 3. The TPC and RSA of fresh sour cherry juice were 1498.58 (mg/L) and 60.84 (%inhibition) at the beginning of the storage period. Our results were similar to those expressed by Yildiz et al. (2022). They evaluated the effect of some treatments on the anthocyanin content of sour cherry juice. According to Table 3, the TPC and RSA of probiotic samples declined significantly by increasing the storage time. This trend can be attributed to the polyphenol oxidase in the pasteurized samples, which oxidizes the phenolic compounds. Also, many other researchers expressed that the bioactive compounds like phenolic compounds decreased by increasing the storage time (Chaikham and Apichartsrangkoon 2012; Sabbaghpour Langaroudi et al. 2023). Nematollahi et al. (2016) investigated the survivability of some probiotic bacteria, RSA, and TPC in cherry juice during refrigerated storage. Similarly, they expressed that the RSA and TPC decreased during cold storage.

**TABLE 3:** Total phenolic content and radical scavenging activity of different sour cherry juice samples.

Property	Storage time (week)		
	0	4	8
Total phenolic content (mg/L)			
Free cell	734.87 ± 2.00^Ac^	727.85 ± 3.90^Ab^	689.26 ± 4.00^Aa^
Alg (1.5%)	1364.59 ± 6.90^Bc^	1315.24 ± 9.30^Bb^	1291.12 ± 2.30^Ba^
Alg:Gel (1.2%:0.3%)	1454.50 ± 11.80^Cc^	1420.51 ± 5.00^Cb^	1348.14 ± 5.00^Ca^
DPPH inhibition (%)			
Free cell	43.23 ± 0.18^Ac^	42.75 ± 0.26^Ab^	42.51 ± 0.25^Aa^
Alg (1.5%)	61.63 ± 0.44^Bc^	60.50 ± 0.18^Bb^	58.47 ± 0.21^Ba^
Alg:Gel (1.2%:0.3%)	61.12 ± 0.80^Bc^	60.96 ± 0.46^Bb^	60.38 ± 0.21^Ca^

*Note:* Different superscripts capital letters in each column and small letters in each row indicate that the means differ significantly (*p <* 0.05). Values are the mean ± SD (*n* = 3).

Abbreviations: Alg, alginate; Gel, gellan fluid gel.

Also, the reduction of TPC and RSA of samples can be related to the presence of probiotic bacteria. Lactobacilli metabolize phenolic compounds by reductases, decarboxylases, and glycosidases (Gaur and Ganzle 2023) and decrease the phenolic content of samples. The most changes were observed in the case of free cells due to more contact with the surrounding media. This phenomenon proved that microencapsulation, especially in the case of alginate/gellan fluid gel beads, reduced the contact of cells with the surrounding media and improved cell viability.

#### 3.3.3 Vitamin C Content

**FIGURE 7:**
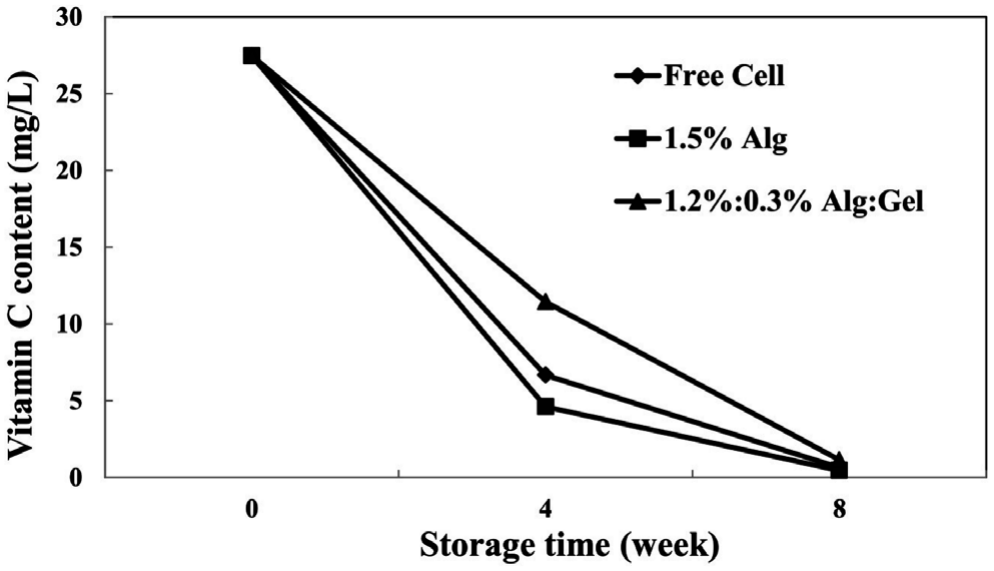
The variation of vitamin C content in different sour cherry juice samples (Alg, alginate; Gel, gellan fluid gel). Mean ± SD (*n* = 3); Statistical test: ANOVA and multiple comparison of means using Duncan's multiple range test (*p* < 0.05).

Vitamin C contents of different sour cherry juice treatments are shown in Figure 7. The results revealed that the vitamin C content of all samples significantly (*p* < 0.05) declined from 27.47 to 0.45–1.14 mg/L during cold storage. The highest amount of vitamin C was observed in the case of alginate/gellan fluid gel beads at the end of the storage period (1.144 mg/L). The reduction rate of vitamin C content depended on the processing and storage temperature. The rapid decline of vitamin C at the start of the storage period can be related to the prompt reaction of ascorbic acid with dissolved oxygen (Polydera et al. 2003). In addition, other studies expressed that the vitamin C content of fresh or probiotic fruit juice declined during cold storage (Plaza et al. 2006; da Mamede Costa et al. 2017).

#### 3.3.4 Viability of *L. reuteri* in Sour Cherry Juice During the Storage Period

**FIGURE 8:**
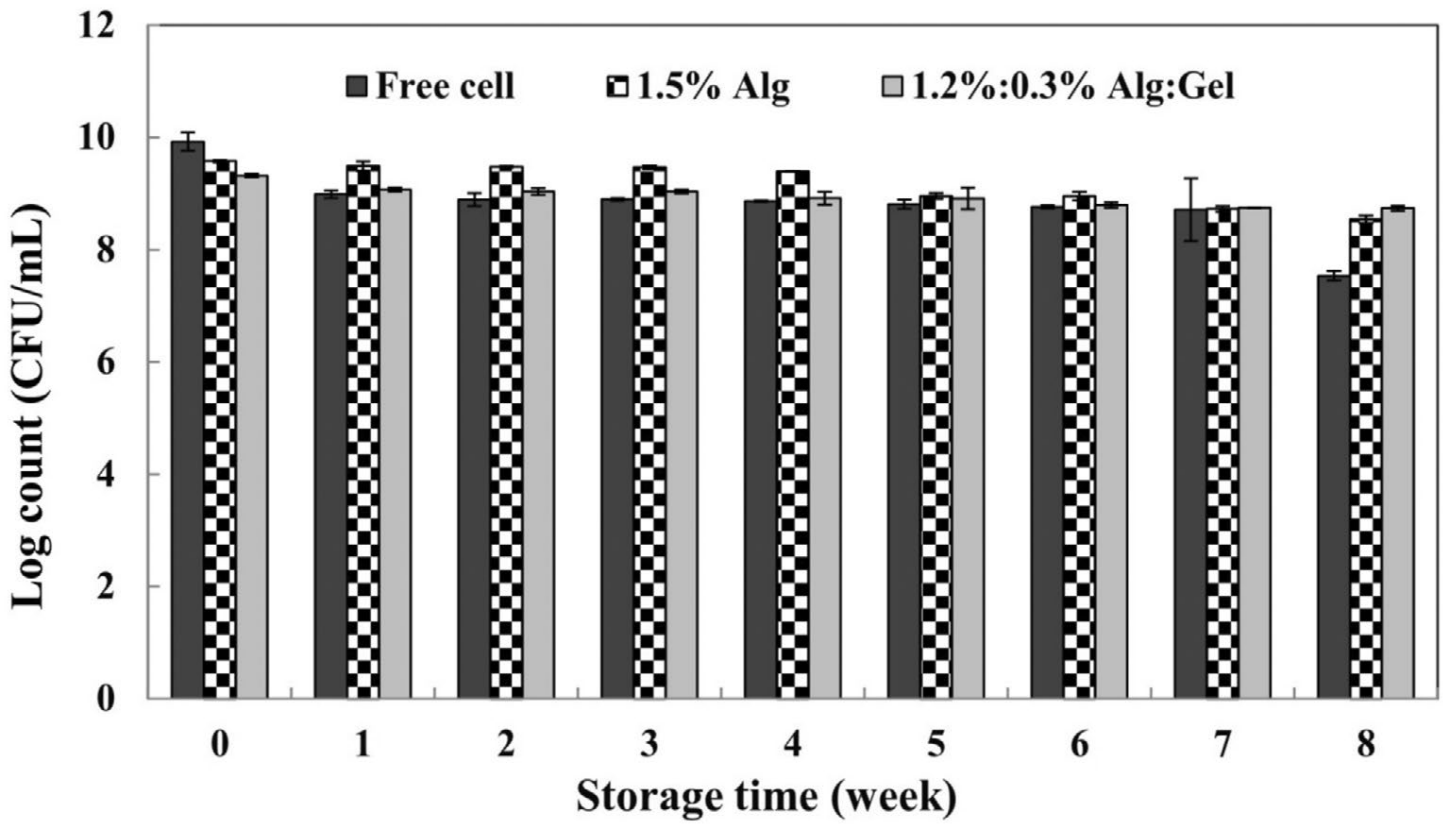
Viability of free and encapsulated *Limosilactobacillus reuteri* (log CFU/mL) in sour cherry juice (4°C) (Alg, alginate; Gel, gellan fluid gel). Mean ± SD (*n* = 3); Statistical test: ANOVA and multiple comparison of means using Duncan's multiple range test (*p* < 0.05).

Figure 8 shows the viability results of free and encapsulated *L. reuteri* during storage at 4°C for 8 weeks. The survivability of probiotics in fruit juices depends on the pH. In the free cell samples, a significant decrease (2.4 log) was observed in the number of viable cells during the storage period, while the lowest change was observed in the case of alginate/gellan fluid gel microcapsule (0.6 log CFU/mL). This issue reveals that encapsulation, especially the alginate/gellan fluid gel system, efficiently protected *L. reuteri* against the surrounding environment and improved the viability of cells. Belyani et al. (2023) investigated the survivability of *L. acidophilus La‐5* encapsulated with *Spirulina platensis* in sour cherry juice. Their results showed a decreasing trend in bacterial death in sour cherry juice containing encapsulated bacteria after 28 days of storage at 4°C. Perricone et al. (2014) evaluated the viability of free *L. reuteri* in different fruit juices and expressed that the survival of free *L. reuteri* was strongly affected by the fruit juice type, with a significant reduction in red fruit, but this problem can be overcome by strain adaptation.

#### 3.3.5 SEM Images of Beads in Sour Cherry Juice During the Storage Period

**FIGURE 9:**
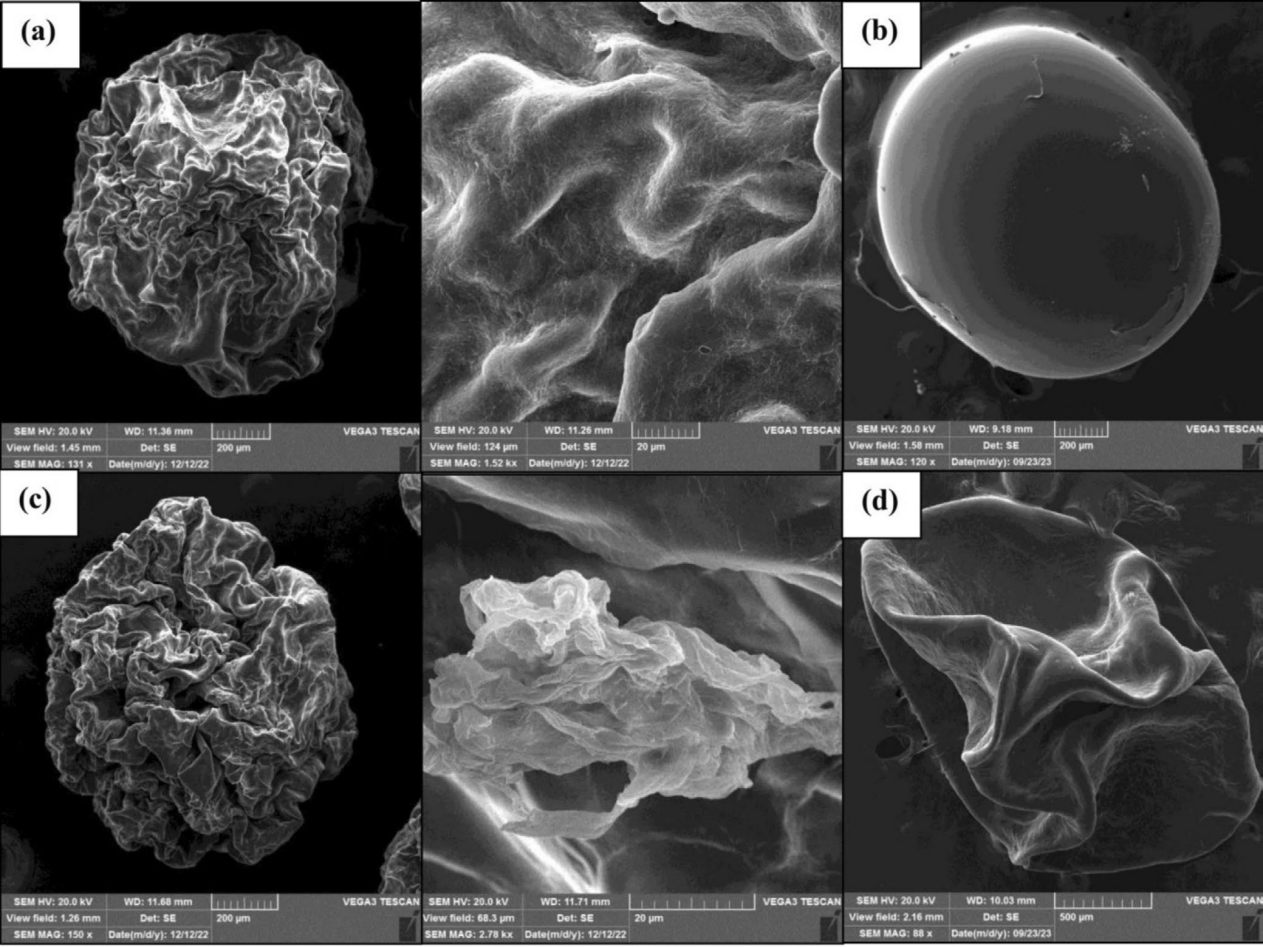
SEM images of (a, b) alginate (1.5%, w/w) and (c, d) alginate:gellan fluid gel (1.2%:0.3%, w/w) beads before (a, c) and after (b, d) of 8 weeks of storage in sour cherry juice (c: shows the particles of gellan fluid gel within the alginate bead).

The morphology characteristics of *L. reuteri* beads were evaluated using the SEM technique. As given in Figure 9a,c, the alginate and alginate/gellan fluid gel beads were moderately spherical. The spherical shape facilitates consumption, industrial production, and packaging of the beads. The alginate and alginate/gellan fluid gel beads showed different morphologies. Figure 9c shows that the particles of gellan fluid gel are dispersed in the alginate bead. Alginate/gellan fluid gel beads showed a dense structure and a relatively smooth surface, but the 1.5% alginate beads showed more porosity and were wrinkled (Figure 9a). Many studies have confirmed the spherical and porous structure of alginate beads (Brachkova et al. 2010; Liu et al. 2019). Regarding the SEM images of the microcapsules after 8 weeks of storage in sour cherry juice (Figure 9b,d), we can observe that the alginate beads were swelled. This phenomenon can be attributed to the more porous structure of alginate beads than alginate/gellan fluid gel beads and confirms that the alginate/gellan fluid beads efficiently protect *L. reuteri* cells from the surrounding medium.

## 4 Conclusion

This study investigated the combination of gellan fluid gel with the alginate bead system as a carrier for encapsulating *L. reuteri* and its application in sour cherry juice. The results indicated that incorporating gellan fluid gel into alginate beads, especially 1.2%:0.3% of Alg:Gel formulation, resulted in a lower surface area and packed the porous structure of alginate beads. So, this carrier improved probiotic viability under simulated gastrointestinal (up to 7.17 log CFU/mL) and thermal conditions (up to 7.00 log CFU/mL) and storage periods. In addition, this system was adequate for protecting *L. reuteri* in sour cherry juice for 8 weeks of storage, in the optimal range of 10^7^ CFU/mL. This system represents an excellent alternative to protect lactic acid bacteria in harsh conditions such as gastrointestinal tract and food processing conditions so we can apply it to processed foods. Drinks such as fruit juices are more favorable and suitable carriers especially for children's application. However, the application of this system in other fruit juices and drinks and non‐refrigerated foods should be investigated in future studies.

## Author Contributions

**Zahra Najafpour:** conceptualization (equal), data curation (equal), formal analysis (equal), methodology (equal), software (equal), validation (equal), visualization (equal), writing – original draft (equal). **Mohammad‐Taghi Golmakani:** conceptualization (equal), funding acquisition (equal), project administration (equal), resources (equal), software (equal), supervision (equal), validation (equal), writing – review and editing (equal). **Marzieh Moosavi‐Nasab:** funding acquisition (equal), project administration (equal), resources (equal), supervision (equal), validation (equal). **Seyed Mohammad Hashem Hosseini:** supervision (equal). **Mehrdad Niakousari:** supervision (equal).

## Conflicts of Interest

The authors declare no conflicts of interest.

## Data Availability

The data that support the findings of this study are available from the corresponding authors upon reasonable request.
